# Epidemiology, treatment, and survival in small cell lung cancer in Spain: Data from the Thoracic Tumor Registry

**DOI:** 10.1371/journal.pone.0251761

**Published:** 2021-06-02

**Authors:** Fernando Franco, Enric Carcereny, Maria Guirado, Ana L. Ortega, Rafael López-Castro, Delvys Rodríguez-Abreu, Rosario García-Campelo, Edel del Barco, Oscar Juan, Francisco Aparisi, Jose L. González-Larriba, Manuel Domine, Jose M. Trigo, Manuel Cobo, Sara Cerezo, Julia Calzas, Bartomeu Massutí, Joaquim Bosch-Barrera, Paola García Coves, Marta Domènech, Mariano Provencio

**Affiliations:** 1 Hospital Universitario Puerta de Hierro-Majadahonda, Madrid, Spain; 2 Catalan Institute of Oncology, Hospital Universitari Germans Trias i Pujol, B-ARGO, IGTP, Badalona, Spain; 3 Hospital General Universitario de Elche, Elche, Spain; 4 Hospital Universitario de Jaén, Jaén, Spain; 5 Hospital Clínico Universitario de Valladolid, Valladolid, Spain; 6 Hospital Universitario Insular de Gran Canaria, Las Palmas de Gran Canaria, Spain; 7 Complejo Hospitalario Universitario A Coruña, A Coruña, Spain; 8 Hospital Universitario de Salamanca, Salamanca, Spain; 9 Hospital Universitario y Politécnico La Fe, Valencia, Spain; 10 Hospital General de Valencia, Valencia, Spain; 11 Hospital Universitario Clínico San Carlos, Madrid, Spain; 12 Hospital Universitario Fundación Jiménez Díaz, IIS-FJD, Madrid, Spain; 13 Unidad de Gestión Clínica Intercentros de Oncología Médica, Hospitales Universitarios Regional y Virgen de la Victoria, IBIMA, Málaga, Spain; 14 Hospital General La Mancha Centro, Alcázar de San Juan, Spain; 15 Hospital Universitario de Fuenlabrada, Fuenlabrada, Spain; 16 Hospital General Universitario de Alicante, Alicante, Spain; 17 Catalan Institute of Oncology, Girona, Spain; University of Nebraska Medical Center, UNITED STATES

## Abstract

**Background:**

Small-cell lung cancer (SCLC) is an aggressive disease with high metastatic potential and poor prognosis. Due to its low prevalence, epidemiological and clinical information of SCLC patients retrieved from lung cancer registries is scarce.

**Patients and methods:**

This was an observational multicenter study that enrolled patients with lung cancer and thoracic tumors, recruited from August 2016 to January 2020 at 50 Spanish hospitals. Demographic and clinical data, treatment patterns and survival of SCLC patients included in the Thoracic Tumor Registry (TTR) were analyzed.

**Results:**

With a total of 956 cases, the age of 64.7 ± 9.1 years, 78.6% were men, 60.6% smokers, and ECOG PS 0, 1 or ≥ 2 in 23.1%, 53.0% and 23.8% of cases, respectively. Twenty percent of patients had brain metastases at the diagnosis. First-line chemotherapy (CT), mainly carboplatin or cisplatin plus etoposide was administered to >90% of patients. In total, 36.0% and 13.8% of patients received a second and third line of CT, respectively. Median overall survival was 9.5 months (95% CI 8.8–10.2 months), with an estimated rate of 70.3% (95% CI 67.2–73.4%), 38.9% (95% CI 35.4–42.4%), and 14.8% (95% CI 11.8–17.8%) at 6, 12 and 24 months respectively. Median progression-free survival was 6.3 months. Higher mortality and progression rates were significantly associated with male sex, older age, smoking habit, and ECOG PS 1–2. Long-term survival (> 2 years) was confirmed in 6.6% of patients, showing a positive correlation with better ECOG PS, poor smoking and absence of certain metastases at diagnosis.

**Conclusion:**

This study provides an updated overview of the clinical situation and treatment landscape of ES-SCLC in Spain. Our results might assist oncologists to improve current clinical practice towards a better prognosis for these patients.

## Introduction

Lung cancer remains a major public health issue worldwide, with more than 2 million new cases and 1.8 million deaths estimated in 2018 [1]. Although smoking is known to be the primary risk factor for lung cancer, other factors such as asbestos, radiation, radon gas and environmental pollution have been also identified [2,3]. While non-small cell lung cancer (NSCLC) represents the majority of diagnosed lung cancer cases (80%), small-cell lung cancer (SCLC) occurs in approximately 15% of patients [4]. Nonetheless, SCLC is the most aggressive subtype of lung cancer, with a 5-year survival rate of below 7% [5].

SCLC is characterized by a rapid doubling time, high growth fraction, early development of widespread metastases, and endocrine paraneoplastic syndromes [6]. Patients usually present shortly after developing symptoms, and metastatic disease appears in approximately 65% of cases [7]. Moreover, nearly 70% of patients are diagnosed with advanced, i.e. extensive stage (ES), disease [8]. Comorbidities are very common and constitute an increasing burden among SCLC patients, often impacting prognosis negatively [9]. Its prevalence seems to increase with age and the presence of multimorbidity has been associated with poorer outcomes [9]. Older age, lower body mass index, poor performance status, ES disease, and best supportive care have emerged as predictors of mortality among SCLC patients [10].

Current therapeutic options for SCLC are limited and there is an unmet need for novel effective treatments to improve survival. Given the aggressive nature of the disease, first-line (1L) platinum-based chemotherapy (CT) has been the mainstay for both limited stage (LS) and ES-SCLC; cisplatin and carboplatin are the most recommended platinum agents in combination with etoposide [6] Recently, the addition of immunotherapy to platinum-etoposide has led to a significant increase in overall survival, thereby constituting the new standard of care in ES-SCLC [6]. While thoracic radiation therapy (RT) along with CT is also recommended to improve local control and survival in LS-SCLC patients [11], consolidative thoracic RT may be considered in selected cases of ES-SCLC who achieved complete response after CT [12,13]. Although 1L CT frequently results in high initial response rates, prognosis remains poor and most ES-SCLC patients experience early disease progression or recurrence [14].

The representation of SCLC patients in lung cancer registries is scarce and ranges between 13.5% and 19% [10,15]. Hence, the main evidence on epidemiological and clinical data of SCLC is often retrieved from hospital- or population-based studies [16–20]. The Thoracic Tumor Registry (TTR) is an observational, multicenter study of lung cancer and other thoracic tumors that was created by the Spanish Lung Cancer Group (GECP) in 2016 with the aim of collecting and unifying population-based data at a national level [21]. In a recent retrospective analysis, epidemiological and clinical data of 6,600 patients with NSCLC were reported [22]. Herein we present a descriptive analysis of the ES-SCLC population included in the TTR up to January 2020.

## Methods

### Study design and sponsor

Observational, prospective, registry-based study that enrolled patients with lung cancer and other thoracic tumors from August 2016 to date. The study was conducted in accordance with the Declaration of Helsinki. Protocol approval was obtained from the institutional review board of Hospital Universitario Puerta de Hierro-Majadahonda (No. PI 148/15). The registry was approved by the Spanish Agency for Medicines and Medical Devices (AEMPS) in 2016, and is registered in the ClinicalTrials.gov database (NCT02941458) [21].

The TTR-2 study was sponsored by the GECP, an independent, multidisciplinary oncology group that coordinates more than 400 experts and 160 hospitals across the Spanish territory. The registry creation was proposed by the steering committee with the aim of promoting lung cancer research and incorporating treatment advances into clinical practice.

### Eligibility

Patients with histologically confirmed lung cancer or other types of thoracic tumors (NSCLC, SCLC, mesothelioma, thymic carcinoma, carcinoid cancer) were eligible for inclusion, without sex or age restrictions. Patients receiving active treatment or palliative care were included. Exclusion criteria were diagnosis of other types of tumors and healthy volunteers. All patients provided signed informed consent before their data were included in the TTR.

### Information retrieval

Data were collected by research teams from patient medical records using an electronic data capture system (EDC). Sociodemographic, epidemiological, clinical, molecular and treatment outcome variables were recorded in an electronic case report form (eCRF). The information was classified into the following categories: (I) patient personal history, which included performance status (PS), tobacco use, and comorbidities; (II) diagnosis, including histological subtype, TNM classification of the tumor and location of metastases; (III) molecular profiling of the tumor; (IV) treatment patterns (surgery, CT, RT); (V) response and survival, including response rates, overall survival and progression-free survival (PFS); and (VI) prognostic factors.

### Statistical analysis

A descriptive statistical analysis was performed. Quantitative variables are presented as mean, standard deviation (SD), median, interquartile range (IQR), minimum, and maximum. Qualitative variables are described as frequencies in the entire population and percentages. Pearson’s chi-square tests were used to compare patient characteristics according to the drug used in first-line CT (carboplatin vs. cisplatin), using Fisher’s exact tests when possible. The OS curve and PFS curves were estimated using the Kaplan-Meier method, evaluating the effect of different characteristics on diagnosis by adjusting univariate Cox regression models. The SPSS IBM Statistics 22 program was used for analysis, setting a significance level of 5% in all bilateral contrast tests. Comparison of 2-year OS rates was performed using the Fixpoint test (package ComparisonSurv of R, method cloglog), as previously described [23]. The Bonferroni correction was applied for multiple comparisons.

## Results

### Patient characteristics

At data cut-off (23^rd^ January 2020), 1,658 (12.9%) SCLC patients were registered in the TTR database, which included a total of 12,867 patients. Of these, 1,037 (62.6%) patients had extensive-stage SCLC, 606 (36.6%) had limited-stage SCLC and 15 (0.9%) had unknown-stage SCLC. Based on data quality and availability, 956 patients with ES-SCLC were finally selected to perform this analysis and their characteristics are shown in Table 1.

**Table 1:** Patient characteristics at baseline (n = 956).

Characteristic	n	%
**Sex**		
Male	751	78.6
Female	205	21.4
**Age at diagnosis**		
Mean (SD), years	64.7 (9.1)	
Median [min-max], years	65 [37–88]	
Distribution		
<55 years	117	12.2
55–64 years	355	37.1
65–74 years	335	35
≥75 years	149	15.6
**Race**		
Caucasian	929	97.2
Other	27	2.8
Patient cancer history*	110	11.5
Head and neck	21	2.2
Bladder/urinary tract	21	2.2
Prostate	14	1.5
Non-melanoma skin	10	1.0
**Smoking habit**		
Never smoker	14	1.5
Former smoker (> 1-year)	357	37.3
Smoker	579	60.6
Unknown	6	0.6
**ECOG at diagnosis**		
0	221	23.1
1	507	53.0
≥2	228	23.8
**Symptoms at diagnosis**		
Asymptomatic	54	5.6
Symptomatic	882	92.3
Unknown	23	2.4
**Metastasis at diagnosis**	924	96.7
Liver	422	44.1
Bone	333	34.8
Thoracic lymphadenopathy	299	31.3
Lung	237	24.8
Extrathoracic lymphadenopathy	206	21.5
Adrenal	203	21.2
CNS	189	19.8
Comorbidities*	826	86.4
Hypertension	460	48.1
Dyslipidemia	330	34.5
COPD	248	25.9
Diabetes mellitus	248	25.9
Heart disease	180	18.8

CNS, central nervous system; COPD, chronic obstructive pulmonary disease; ECOG, Eastern Cooperative Oncology Group.

*The most frequent previous tumor locations and current comorbidities are shown.

Most patients (78.6%) were men, 60.6% smokers, with a mean age of 64.7 ± 9.1 years and ECOG PS 0, 1 or ≥ 2 in 23.1%, 53.0% and 23.8% of cases, respectively. According to the TNM classification (S1 Table) and the AJCC equivalence (8^th^ edition) [24], most patients were diagnosed with stage IVA/B disease. Up to 88.5% did not have any previous cancer history, but the majority of patients presented symptoms at diagnosis, such as cough (39.4%), pain (36.7%), dyspnea (35.6%), and weight loss (28.6%). Mean time between developing symptoms and diagnosis was 1.09 ± 1.98 months. Metastases were observed in almost the entire population (96.7%), 19.8% of which corresponded to brain metastases. Comorbidities appeared in 86.4% of patients, the most common being hypertension, dyslipidemia, chronic obstructive pulmonary disease, and diabetes mellitus. Although molecular profiling of tumors was performed in a very low number of patients, positive results were observed for some biomarkers such as Ki67, TTF1, synaptophysin, enolase, and CD-56 (S2 Table).

### Treatments and response

Mean time between diagnosis and first treatment was 0.68 ± 1.63 months. CT alone or in combination with RT was the treatment of choice in 415 (43.4%) and 449 (47.0%) patients, respectively; RT was mainly provided with palliative (30.9%) and cranial prophylactic (9.4%) intent (Table 2). Patients who were treated with CT and RT, received it sequentially and not concurrently treatment. All of them were cases selected by their oncologist after showing a good response to CT treatment.

**Table 2:** Treatment characteristics.

Treatment	n	%
**Single treatments**		
Radiotherapy (RT)	18	1.9
Chemotherapy (CT)	415	43.4
Surgery	1	0.1
**Combined treatments**		
RT + CT	449	47
Surgery + RT	2	0.2
Surgery + CT	5	0.5
Surgery + RT + CT	10	0.1
**Surgical intent**		
Diagnostic	7	0.7
Curative	6	0.6
Palliative	5	0.5
**Radiotherapy intent**		
Radical	67	7
Adjuvant	13	1.4
Palliative	295	30.9
Prophylactic	90	9.4
Unknown	14	1.5
**Chemotherapy**		
First line	879	91.9
Monotherapy	19	2.2
Combination of 2 drugs	835	95
Combination of 3 drugs	25	2.8
Second line	344	36
Third line	132	13.8

In contrast, surgery was performed in less than 1% of patients. First-line (1L) CT was used in almost the entire population (91.9%), mostly using a combination of 2 drugs: carboplatin + etoposide (Car + E) (61.8%) or cisplatin + etoposide (Cis + E) (31.7%). In total, 36.0% and 13.8% of patients received a second (2L) and third line (3L) of CT, respectively. Consolidative RT was given to 262/879 (29.8%) patients treated with 1L CT; holocranial (58.8%) and thoracic (29.8%) were the most common irradiated areas.

Of the 879 patients who received 1L CT, 416 (52.7%) received 4 cycles or fewer, while 373 (47.3%) received 5 or more cycles. The mean number of cycles of CT was slightly higher in 1L (4.3) compared to 2L (3.7) and 3L (3.4) (Table 3).

**Table 3:** Chemotherapy treatment and response.

	First line (n = 879)	Second line (n = 344)	Third line (n = 132)
**Number of cycles**			
Mean (sd)	4.3 (1.9)	3.7 (3.3)	3.4 (2.7)
Median [min-max]	4 (1–12)	3 (1–31)	3 [1–16]
**Duration of treatment (months)**			
Mean (sd)	2.86 (1.78)	2.39 (2.67)	1.96 (2.11)
Median [min-max]	3.0 [0–16.1]	1.8 [0–18.9]	1.4 [0–15.2]
**Best response by RECIST 1.1 criteria, n (%)**			
CR	24 (2.7%)	9 (2.6%)	1 (0.8%)
PR	449 (51.5%)	64 (18.7%)	17 (12.9%)
SD	72 (8.2%)	55 (16.0%)	22 (16.7%)
PD	108 (12.3%)	128 (37.3%)	49 (37.1%)
NE	87 (9.9%)	48 (14.0%)	24 (18.2%)
ND	50 (5.7%)	18 (5.2%)	4 (3.0%)

CR, complete response; ND, not determined; NE, not estimated; PD, progressive disease; PR, partial response; sd, standard deviation; SD, stable disease.

Likewise, duration of treatment was reduced in subsequent treatment lines from 2.86 months in 1L to 2.39 in 2L and 1.96 in 3L. The best response rates, mainly partial response (PR), were observed after 1L CT (51.5%), while rates of stable disease (SD) showed a 2-fold increase with 2L and 3L compared to 1L. Conversely, progressive disease (PD) was substantially higher in subsequent lines, being observed in more than one-third of patients.

### Overall survival and progression-free survival

**Fig 1:**
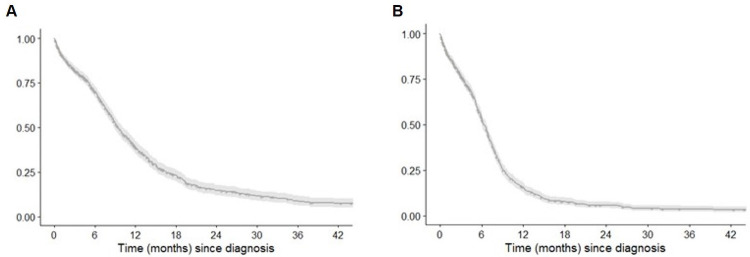
Overall survival (A) and progression-free survival (B) in the study population.

Median OS in the entire population was 9.5 months (95% CI 8.8–10.2 months), with an estimated OS rate of 70.3% (95% CI 67.2–73.4%) at 6 months, 38.9% (95% CI 35.4–42.4%) at 12 months, 26.2% (95% CI 22.8–29.6%) at 18 months, and 14.8% (95% CI 11.8–17.8%) at 24 months after diagnosis (Fig 1A). Greater survival was observed based on the number of CT lines received (*p* < 0.001); however, a selection bias should be noted as patients who received 3 or more lines survived longer. Higher mortality rates were significantly associated with male sex, older age, smoking habit, and ECOG PS 1–2 (S3 Table).

Overall, 649 (67.9%) patients progressed during follow-up, 105 of whom remained alive at the end of the study. Progressions were mostly distant and/or local, often affecting lung, thoracic lymph nodes, and liver. Median PFS was 6.3 months (95% CI 6.0–6.7 months), with an estimated PFS rate of 53.8% (95% CI 50.4–57.2%) at 6 months, 15.6% (95% CI 13.0–18.3%) at 12 months, 7.9% (95% CI 5.9–9.9%) at 18 months, and 5.8% (95% CI 4.0–7.6%) at 24 months (Fig 1B). As described for OS, sex, age, smoking and ECOG PS at diagnosis were factors significantly associated with poorer PFS outcomes (S4 Table).

### Patients treated with Car/Cis + E in 1L

In total, 543 patients were treated with Car + E and 279 with Cis + E in 1L. S5 Table shows the distribution of patients according to their characteristics at diagnosis. A significantly higher proportion of women, younger age, better ECOG PS, and lower percentage of liver metastasis were observed among patients who received cisplatin. A similar distribution of brain metastases was observed between subgroups.

The number of cycles and duration of treatment did not differ among patients treated with Car + E or Cis + E (S6 Table). However, the latter showed a higher rate of response (complete or partial) and SD, with a lower proportion of patients who progressed. Overall, 703/822 (85.5%) patients finished 1L treatment, of whom 314 (44.7%) started a subsequent 2L: 193 (41.3%) with Car + E and 121 (51.3%) with Cis + E. Mean time from end of 1L to start of 2L CT ranged from 4.42 to 4.95 months.

#### Survival in patients treated with Car/Cis + E

**Fig 2:**
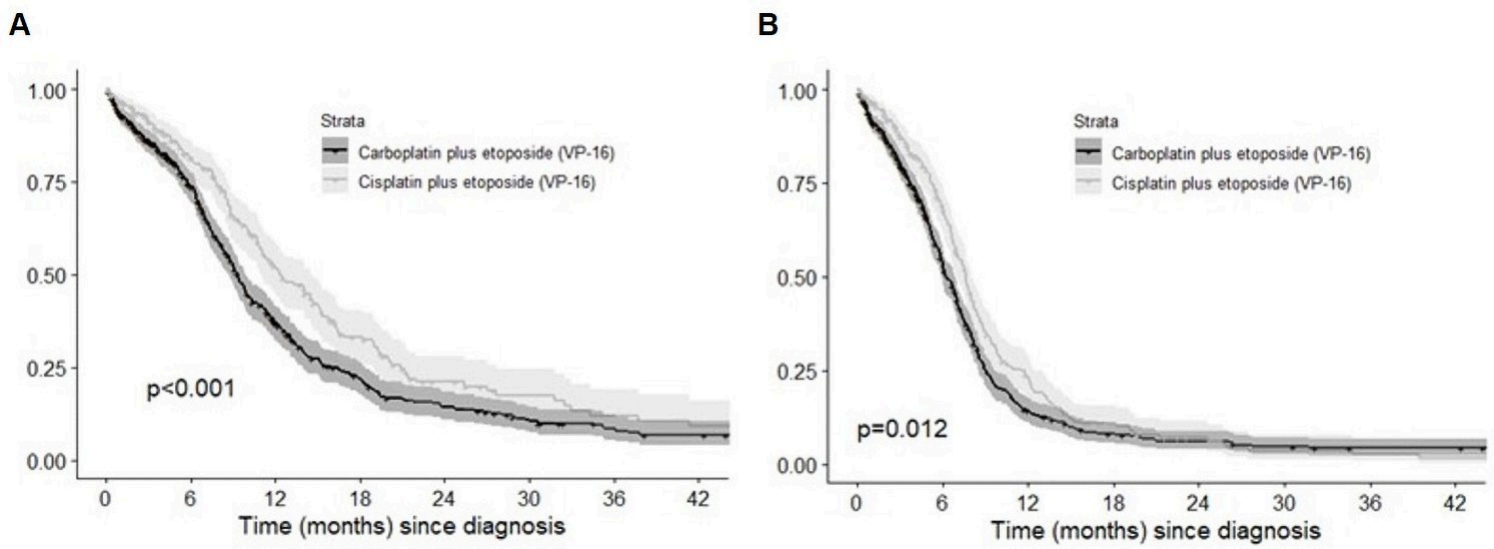
Overall survival (A) and progression-free survival (B) in patients treated with first-line carboplatin/cisplatin + etoposide VP16.

Of the 543 patients with Car + E, 366 (67.4%) died during follow-up, with a median OS of 9.3 months (95% CI 8.7–9.9 months) since diagnosis. A significantly lower rate of mortality was observed among patients treated with Cis + E (162/279, 58.1%), with a higher median OS at 12.5 months (95% CI 10.5–14.6) (p < 0.001) (Fig 2A). Likewise, median PFS was significantly higher among patients who received Cis + E (7.6 months [95% CI 7.1–8.2 months]) than those treated with Car + E (6.2 months [95% CI 5.7–6.7 months]) (p = 0.012) (Fig 2B). Differences among subgroups were also significant when OS was estimated from the start of 1L treatment: 11.5 (9.8–13.2) months in Cis + E and 8.8 (8.3–9.4) months in Car + E patients (p < 0.001). In total, 109/703 (15.5%) patients remained alive without a 2L treatment. Median survival of patients treated with 1L Car + E or Cis + E was 3.1 months (95% CI 2.7–3.5 months) and 3.9 months (95% CI 3.4–4.3 months), respectively.

### Long-term survival

In total, 63/956 (6.6%) patients (44 men and 19 women) were alive after a 2-year follow-up. Most (95.2%) were smokers or former smokers and presented ECOG PS 0 (31.7%) or 1 (58.7%) and metastasis at diagnosis, liver (30.2%), bone (23.8%) and lung (22.2%) being the most frequent locations (S7 Table). A higher proportion of patients received Car + E than Cis + E (63.5% and 31.7%, respectively) as 1L treatment. Multivariate analysis of 2-year OS rates considering patient characteristics at diagnosis is shown in Table 4.

**Table 4:** Multivariate analysis of 2-year OS rates according to patient characteristics.

	2-year OS rate (%)	95% CI	*p* value
**Sex**			0.077
Male	13.4	10.5–17.1	
Female	20.3	14.1–29.0	
**Age**			0.117
<65 years	17.2	12.9–21.9	
≥65 years	12.4	8.8–16.6	
**ECOG**			0.001
0	23	15.9–30.9	
1	14.9	11.1–19.2	
≥2	6.7	3.3–11.8	
**Smoking habit**	20.7	15.6–26.3	0.004
Never/former smoker	11.5	8.3–15.2	
Smoker			
**Metastasis**			
Liver	9.2	5.9–13.3	0.001
Bone	9.7	6.1–14.4	0.012
Thoracic adenopathy	9.6	5.5–15.1	0.031
Lung	14.3	8.8–21.0	0.85
Extrathoracic adenopathy	13.3	7.9–20.0	0.594
Adrenal	10.6	6.1–16.5	0.117
CNS	11.9	6.4–19.3	0.367
Pleural effusion	11.8	6.2–19.3	0.36
**Treatment**			0.078
Carboplatin + Etoposide VP16	14.3	10.8–18.4	
Cisplatin + Etoposide VP16	21	14.8–28.0	

CI, confidence interval; CNS, central nervous system; ECOG, Eastern Cooperative Oncology Group; OS, overall survival.

A significantly greater long-term survival rate was associated with better ECOG PS, poor smoking habit, and the absence of liver, bone, and thoracic lymph node metastasis. Conversely, factors such as sex, age, other metastatic locations, or treatment failed to show statistical differences among this population.

## Discussion

Following the recent publication of the TTR NSCLC population report [22], we analyzed the epidemiological and clinical data of 956 patients diagnosed with ES-SCLC who were recruited at 50 Spanish hospitals up to January 2020. To our knowledge, this is the first nationwide study providing an accurate description of the current situation, treatment patterns, and survival outcomes of SCLC patients. As reported in other registries [10,15], we identified more than 60% of patients with ES-SCLC (stage IV) among the Spanish SCLC population registered in the TTR. Most of these patients presented symptoms, metastases, and comorbidities at diagnosis, and had no previous history of cancer. In accordance with clinical guidelines for SCLC management [6,25], 1L CT alone or in combination with RT was administered to almost the entire population. With high progression and mortality rates, poor survival outcomes and associated negative prognostic factors were in line with reported evidence on this type of patients [6,10,14,18–20].

Cis + E has remained the standard of care for ES-SCLC patients for decades, while Car + E is alternatively advised in case of contraindications for Cis (level of evidence I, degree of recommendation A) [6]. Although the optimal duration of CT in these patients is not well defined, a maximum of 4–6 cycles is usually recommended [6]. Among our study population, a higher number of patients were treated with Car + E than Cis + E, and approximately half received 4 or fewer cycles of 1L CT. A systematic review of four randomized controlled trials (RCTs) compared Cis with Car + E in 1L treatment of SCLC, reporting no statistical differences in median OS (9.6 vs 9.4 months), median PFS (5.5 vs. 5.3 months), and overall response (67% vs. 66%) [26]. However, the safety profile differed between the treatments, with a higher hematologic toxicity being associated with Car and higher non-hematologic toxicity related to Cis [26]. Recently, these findings were corroborated in a meta-analysis of 12 RCTs, suggesting that treatment choice should be based on the toxicity profile of Cis/Car alongside patient’s comorbidities [27]. Interestingly, our study revealed that patients treated with Cis + E had higher response rates, fewer progressions, and significantly greater median OS and PFS compared to those who received Car + E. Nevertheless, it should be noted that these differences might be related more with the more favorable profile of ES-SCLC patients treated with Cis + E than with the treatment itself. Several factors have been identified as negative predictors of mortality in SCLC patients. Older age, male sex, poor ECOG PS, larger tumor size, multiple metastatic sites and increased creatinine levels are associated with poor prognosis of ES-SCLC disease [10,18,20]. Among the TTR SCLC population, patients who received Cis + E included a higher proportion of women, of a younger age, and with better ECOG PS and fewer liver metastasis than those treated with Car + E.

RT for thoracic lesions and metastatic sites, except for brain metastases, has been associated with improved OS and cancer-specific survival in patients with metastatic ES-SCLC [28]. As such, the Spanish Society of Medical Oncology (SEOM) recommends that consolidative thoracic RT should be considered in selected patients with ES-SLCL who have completed CT and achieved complete or near complete response, especially in patients with good extrathoracic response (I, B) [6]. In this study, RT was provided with palliative intent in nearly one-third of the population and consolidative RT, mostly holocranial, was given to nearly 30% of those treated with 1L CT. With a reported 20% incidence among this study population, it is estimated that approximately half of SCLC patients will develop brain metastases within 2 years after diagnosis [29]. Given that prophylactic cranial irradiation (PCI) has been shown to decrease the development of brain metastases by 25% [30], this strategy is advisable in ES-SCLC patients who respond to primary CT [6,29]. The roll of PCI in ES-SCLC was evaluated in a Japanese clinical trial with 224 patients (randomization 1:1). Median OS was 11.6 months (95% CI 9.5–13.3) in the first group versus 13.7 months in the control arm (10.2–16.4), HR 1, 27, 95% CI 0.96–1.68; p = 0.094. In our study, a small percentage of patients received PCI. Accordingly, the SEOM guidelines established that PCI should be evaluated in patients with good PS who achieve a response (I, B) [6]. Although more than 50% of SCLC patients showed CR/PR in our study, prophylactic RT was given to less than 10% of the population.

In line with previous studies, particularly those which included ES-SCLC patients [6,15,18], low survival and high progression rates were found among the TTR SCLC population. Median OS and PFS barely reached 9.5 months and 6.3 months, respectively, after 1L CT; male sex, older age, smoking habit and ECOG PS 1–2 were significantly associated with worse prognosis. Noticeably, evidence on survival trends shows an improvement in the prognosis of SCLC patients in the last decades. A previous analysis demonstrated significantly improved overall and stage-specific median survival times and 5-year survival rates of 1,032 SCLC patients treated at the Moffitt Cancer Center from 1986 to 2008 [19]. While the 5-year OS rate significantly increased from 8.3% to 11.0%, the median OS increased from 11.3 months to 15.2 months [19]. More recently, a study among the Japanese population reported that 5-year relative survival of 10,911 LS- and ES-SCLC patients significantly increased from 1999–2006 compared to 1993–1998 [16]. To date, our results indicate an estimated 2-year OS rate of 14.8% and PFS rate of 5.8% among the TTR SCLC Spanish population monitored since 2016. Furthermore, long-term survival (> 2 years), which was associated with better ECOG PS, poor smoking habit and absence of certain metastases at diagnosis, has been confirmed in 6.6% of patients. Close monitoring of these patients over a longer follow-up will help discern survival trends and potential improvements due to the implementation of novel therapies in current clinical practice.

Based on the encouraging results from phase III trials, the combination of CT and immunotherapy has been recently established as the first-line treatment of adult patients with ES-SCLC (I, A) [6]. In the IMpower133 trial, atezolizumab in combination with Car + E showed a significant benefit in median OS (12.3 vs. 10.3 months; HR 0.70) and OS rate at 18 months (34% vs. 21%) compared to Car + E [31]. This regimen, therefore, was first approved by the Food and Drug Administration (FDA), and later by the European Medicines Agency (EMA) in 2019. The updated data from this study were presented at ESMO 2020. With a follow-up of 22.9 months, OS at 24 months was 22% vs 16.8% in favor of the experimental arm (atezolizumab plus CT) [32]. On the other hand, the combination of durvalumab with Cis/Car + E is also a recommended treatment option for ES-SCLC (I, A) [6], as it demonstrated a significant improvement in median OS (13.0 vs. 10.3 months; HR 0.73) compared to CT in the CASPIAN trial [33]. In light of these findings, real-world evidence studies are warranted to confirm and further explore clinical benefits of checkpoint inhibition in this setting.

The main limitations of this study stem from its observational, retrospective design. Despite the potential bias in the recruited population among the participant centers, the sample size was large enough to provide an objective nationwide epidemiological overview of ES-SCLC status. Moreover, all patients had equal opportunities for diagnosis and treatment, as established by the universal coverage of the Spanish National Health System, and they were enrolled in the study over a short time period, thereby enabling a more reliable comparison of therapies and survival.

## Conclusions

This study provides an accurate overview of the current clinical situation and treatment landscape of ES-SCLC in Spain. With a high proportion of patients diagnosed with metastatic disease and a very poor prognosis, epidemiological data and survival outcomes of this population are in line with those reported by previous studies across different countries. Since these results support current evidence on the aggressiveness of ES-SCLC and the need for more effective therapies, the development of further studies is warranted. Continuous monitoring of TTR data will help evaluate the impact of current and novel treatments used in clinical practice, with the aim of eventually improving the prognosis and survival of SCLC patients.

## Supporting information

S1 Table Patient distribution according to TNM stage. (DOCX)

S2 Table Molecular profiling of tumors at diagnosis. ALK, anaplastic lymphoma kinase; BRAF, B-RAF proto-oncogene, serine/threonine kinase oncogene; EGFR, epidermal growth factor receptor; FISH, fluorescence in situ hybridization; FGFR1, fibroblast growth factor receptor type 1; HER2, human epidermal growth factor receptor type 2; IHC, immunohistochemistry; KRAS, Kirsten rat sarcoma viral oncogene homolog; MET, tyrosine-protein kinase MET/hepatocyte growth factor receptor; PD-L1, programmed death-ligand 1; RET, proto-oncogene tyrosine-protein kinase receptor; RNA, ribonucleic acid; ROS1, ROS proto-oncogene 1 receptor tyrosine kinase; TTF1, thyroid transcription factor 1.
(DOCX)

S3 Table Overall survival according to demographic and diagnostic factors. CNS, central nervous system; ECOG, Eastern Cooperative Oncology Group; HR, hazard ratio; CI, confidence interval; SD, standard deviation.
(DOCX)

S4 Table Progression-free survival according to demographic and diagnostic factors. CNS, central nervous system; ECOG, Eastern Cooperative Oncology Group; HR, hazard ratio; CI, confidence interval; SD, standard deviation.
(DOCX)

S5 Table Characteristics of patients treated with carboplatin/cisplatin + etoposide VP16 first line chemotherapy. CNS, central nervous system; ECOG, Eastern Cooperative Oncology Group.
(DOCX)

S6 Table Characteristics of carboplatin/cisplatin + etoposide VP16 first line treatment and response. CR, complete response; PR, partial response; sd, standard deviation; SD, stable disease; PD, progressive disease; NE, not estimated; ND, not determined.
(DOCX)

S7 Table Patient characteristics of long-term survivors (> 2 years; n = 63). CNS, central nervous system; ECOG, Eastern Cooperative Oncology Group.
(DOCX)
