# Supplementary material for: Epidemiology, treatment, and survival in small cell lung cancer in Spain: Data from the Thoracic Tumor Registry
Source: PLoS One. 2021 Jun 2;16(6):e0251761. doi: 10.1371/journal.pone.0251761 (PMC8171958; doi:10.1371/journal.pone.0251761)
Supplement: S1 Table — (DOCX) [file pone.0251761.s001.docx]

**S1 Table.** **Patient distribution according to TNM stage.**

|  | n | % |
| --- | --- | --- |
| Stage T  Tx  T0  T1  T2  T3  T4 | 218  2  69  116  130  421 | 22.8  0.2  7.2  12.1  13.6  44.0 |
| Stage N  Nx  N0  N1  N2  N3 | 220  51  35  270  380 | 23.0  5.3  3.7  28.2  39.7 |
| Stage M  Mx  M0  M1a  M1b  M1c | 39  16  127  385  389 | 4.1  1.7  13.3  40.3  40.7 |
