# Supplementary material for: Epidemiology, treatment, and survival in small cell lung cancer in Spain: Data from the Thoracic Tumor Registry
Source: PLoS One. 2021 Jun 2;16(6):e0251761. doi: 10.1371/journal.pone.0251761 (PMC8171958; doi:10.1371/journal.pone.0251761)
Supplement: S2 Table — ALK, anaplastic lymphoma kinase; BRAF, B-RAF proto-oncogene, serine/threonine kinase oncogene; EGFR, epidermal growth factor receptor; FISH, fluorescence in situ hybridization; FGFR1, fibroblast growth factor receptor type 1; HER2, human epidermal growth factor receptor type 2; IHC, immunohistochemistry; KRAS, Kirsten rat sarcoma viral oncogene homolog; MET, tyrosine-protein kinase MET/hepatocyte growth factor receptor; PD-L1, programmed death-ligand 1; RET, proto-oncogene tyrosine-protein kinase receptor; RNA, ribonucleic acid; ROS1, ROS proto-oncogene 1 receptor tyrosine kinase; TTF1, thyroid transcription factor 1. (DOCX) [file pone.0251761.s002.docx]

**S2 Table. Molecular profiling of tumors at diagnosis.**

| Biomarker | n | % |
| --- | --- | --- |
| Total | 956 | 100.0 |
| EGFR  Negative  Positive  T790M(+)  T790M(-)  Exon 19  Exon 21  NOS  Other type | 19  17  2  0  0  0  2  0  0 | 2.0  1.8  0.2  0.0  0.0  0.0  0.2  0.0  0.0 |
| ALK (IHC)  Negative  Positive | 9  9  0 | 0.9  0.9  0.0 |
| ALK (FISH)  No translocation  Translocation | 3  3  0 | 0.3  0.3  0.0 |
| ALK (RNA) | 0 | 0.0 |
| KRAS  Not detected  Detected | 5  4  1 | 0.5  0.4  0.1 |
| HER2 (IHC) | 0 | 0.0 |
| HER2 (FISH) | 0 | 0.0 |
| HER2 (mutated)  Not detected  Detected | 2  2  0 | 0.2  0.2  0.0 |
| ROS (HQ)  Negative  Positive | 10  10  0 | 1.0  1.0  0.0 |
| ROS (FISH)  No translocation  Translocation | 4  4  0 | 0.4  0.4  0.0 |
| FGFR1 | 0 | 0.0 |
| PD-L1  Not provided  Negative  Positive | 13  1  8  4 | 1.4  0.1  0.8  0.4 |
| RET | 0 | 0.0 |
| DLL3  Negative  Positive | 2  0  2 | 0.2  0.0  0.2 |
| BRAF  Not detected  Detected | 5  4  1 | 0.5  0.4  0.1 |

**S2 Table 2 (cont.). Molecular profiling of tumors at diagnosis.**

| Biomarker | n | % |
| --- | --- | --- |
| MET  Negative  Amplified  Overexpressed  Mutated | 2  2  0  0  0 | 0.2  0.2  0.0  0.0  0.0 |
| Ki67  Negative  Positive | 4  0  4 | 0.4  0.0  0.4 |
| TTF1  Negative  Positive | 11  2  9 | 1.2  0.2  0.9 |
| Synaptophysin  Negative  Positive | 11  0  11 | 1.2  0.0  1.2 |
| Enolase  Negative  Positive | 3  0  3 | 0.3  0.0  0.3 |
| CD-56  Negative  Positive | 7  0  7 | 0.7  0.0  0.7 |
| Other tests | 13 | 1.4 |

ALK, anaplastic lymphoma kinase; BRAF, B-RAF proto-oncogene, serine/threonine kinase oncogene; EGFR, epidermal growth factor receptor; FISH, fluorescence in situ hybridization; FGFR1, fibroblast growth factor receptor type 1; HER2, human epidermal growth factor receptor type 2; IHC, immunohistochemistry; KRAS, Kirsten rat sarcoma viral oncogene homolog; MET, tyrosine-protein kinase MET/hepatocyte growth factor receptor; PD-L1, programmed death-ligand 1; RET, proto-oncogene tyrosine-protein kinase receptor; RNA, ribonucleic acid; ROS1, ROS proto-oncogene 1 receptor tyrosine kinase; TTF1, thyroid transcription factor 1.
