# Supplementary material for: Epidemiology, treatment, and survival in small cell lung cancer in Spain: Data from the Thoracic Tumor Registry
Source: PLoS One. 2021 Jun 2;16(6):e0251761. doi: 10.1371/journal.pone.0251761 (PMC8171958; doi:10.1371/journal.pone.0251761)
Supplement: S3 Table — CNS, central nervous system; ECOG, Eastern Cooperative Oncology Group; HR, hazard ratio; CI, confidence interval; SD, standard deviation. (DOCX) [file pone.0251761.s003.docx]

**S3 Table. Overall survival according to demographic and diagnostic factors.**

|  | Deceased | Censored | HR | CI 95% | *p* value |
| --- | --- | --- | --- | --- | --- |
| Total | 640 (66.9%) | 316 (33.1%) |  |  |  |
| Sex  Male  Female | 516 (68.7%)  124 (60.5%) | 235 (31.3%)  81 (39.5%) | -  0.731 | 0.601-0.891 | 0.002 |
| Age  Mean (SD)  Median [min-max] | 65.2 (9.4)  65 (37-88) | 63.7 (8.4)  64 (42-87) | 1.026 | 1.017-1.036 | <0.001 |
| Smoking habit  Smoker  Former smoker  Never smoker | 401 (69.3%)  227 (63.6%)  7 (50.0%) | 178 (30.7%)  130 (36.4%)  7 (50.0%) | -  0.850  0.399 | 0.722-1.001  0.188-0.845 | 0.012  0.052  0.016 |
| Asbestos exposure  No  Yes | 189 (56.9%)  21 (65.6%) | 143 (43.1%)  11 (34.4%) | -  1.190 | 0.757-1.870 | 0.452 |
| CNS metastasis  No  Yes | 516 (67.3%)  124 (65.6%) | 251 (32.7%)  65 (34.4%) | -  1.153 | 0.947-1.404 | 0.155 |
| ECOG  0  1  ≥2 | 131 (59.3%)  334 (65.9%)  175 (76.8%) | 90 (40.7%)  173 (34.1%)  53 (23.2%) | -  1.301  2.715 | 1.062-1.592  2.160-3.411 | <0.001  0.011  <0.001 |

CNS, central nervous system; ECOG, Eastern Cooperative Oncology Group; HR, hazard ratio; CI, confidence interval; SD, standard deviation.
