# Supplementary material for: Epidemiology, treatment, and survival in small cell lung cancer in Spain: Data from the Thoracic Tumor Registry
Source: PLoS One. 2021 Jun 2;16(6):e0251761. doi: 10.1371/journal.pone.0251761 (PMC8171958; doi:10.1371/journal.pone.0251761)
Supplement: S4 Table — CNS, central nervous system; ECOG, Eastern Cooperative Oncology Group; HR, hazard ratio; CI, confidence interval; SD, standard deviation. (DOCX) [file pone.0251761.s004.docx]

**S4 Table. Progression-free survival according to demographic and diagnostic factors.**

|  | Event (progression) | Censored | HR | CI 95% | *p* value |
| --- | --- | --- | --- | --- | --- |
| Total | 745 (77.9%) | 211 (22.1%) |  |  |  |
| Sex  Male  Female | 598 (79.6%)  147 (71.7%) | 153 (20.4%)  58 (28.3%) | -  0.802 | 0.669-0.960 | 0.016 |
| Age  Mean (SD)  Median [min-max] | 64.9 (9.3)  65 (37-88) | 64.0 (8.4)  64 (42-87) | 1.016 | 1.007-1.024 | <0.001 |
| Smoking habit  Smoker  Former smoker  Never smoker | 460 (79.4%)  275 (76.4%)  8 (57.1%) | 119 (20.6%)  85 (23.6%)  6 (42.9%) | -  0.877  0.363 | 0.754-1.019  0.180-0.732 | 0.006  0.086  0.005 |
| Asbestos exposure  No  Yes | 234 (70.5%)  26 (81.3%) | 98 (29.5%)  6 (18.8%) | -  1.271 | 0.847-1.908 | 0.247 |
| CNS metastasis  No  Yes | 603 (78.6%)  142 (75.1%) | 164 (21.4%)  47 (24.9%) | -  1.068 | 0.889-1.908 | 0.484 |
| ECOG  0  1  ≥2 | 167 (75.6%)  389 (76.7%)  189 (82.9%) | 54 (24.4%)  118 (23.3%)  39 (17.1%) | -  1.212  2.229 | 1.011-1.453  1.807-2.749 | <0.001  0.038  <0.001 |

CNS, central nervous system; ECOG, Eastern Cooperative Oncology Group; HR, hazard ratio; CI, confidence interval; SD, standard deviation.
