# Supplementary material for: Epidemiology, treatment, and survival in small cell lung cancer in Spain: Data from the Thoracic Tumor Registry
Source: PLoS One. 2021 Jun 2;16(6):e0251761. doi: 10.1371/journal.pone.0251761 (PMC8171958; doi:10.1371/journal.pone.0251761)
Supplement: S5 Table — CNS, central nervous system; ECOG, Eastern Cooperative Oncology Group. (DOCX) [file pone.0251761.s005.docx]

**S5 Table. Characteristics of patients treated with carboplatin/cisplatin + etoposide VP16 first line chemotherapy.**

|  | Carboplatin + Etoposide VP16  (n=543) | Cisplatin + Etoposide VP16  (n=279) | *p* value |
| --- | --- | --- | --- |
| Sex  Male  Female | 436 (80.3%)  107 (19.7%) | 201 (72.0%)  78 (28.0%) | 0.008 |
| Age  <55 years  55-64 years  65-74 years  ≥75 years | 56 (10.3%)  185 (34.1%)  210 (38.7%)  92 (16.9%) | 49 (17.6%)  127 (45.5%)  89 (31.9%)  14 (5.0%) | <0.001 |
| ECOG  0  1  ≥2 | 120 (22.1%)  298 (54.9%)  125 (23.0%) | 77 (27.6%)  157 (56.3%)  45 (16.1%) | 0.036 |
| Metastasis  Liver  Bone  Thoracic adenopathy  Lung  Extrathoracic adenopathy  Adrenal  CNS  Pleural effusion | 96 (17.7%)  69 (12.7%)  91 (16.8%)  152 (28.0%)  36 (6.6%)  49 (9.0%)  75 (13.8%)  26 (4.8%) | 33 (11.8%)  33 (11.8%)  45 (16.1%)  82 (29.4%)  22 (7.9%)  21 (7.5%)  31 (11.1%)  11 (3.9%) | 0.033  0.739  0.844  0.684  0.565  0.511  0.323  0.723 |

CNS, central nervous system; ECOG, Eastern Cooperative Oncology Group.
