# Supplementary material for: Epidemiology, treatment, and survival in small cell lung cancer in Spain: Data from the Thoracic Tumor Registry
Source: PLoS One. 2021 Jun 2;16(6):e0251761. doi: 10.1371/journal.pone.0251761 (PMC8171958; doi:10.1371/journal.pone.0251761)
Supplement: S6 Table — CR, complete response; PR, partial response; sd, standard deviation; SD, stable disease; PD, progressive disease; NE, not estimated; ND, not determined. (DOCX) [file pone.0251761.s006.docx]

**S6 Table.** **Characteristics of carboplatin/cisplatin + etoposide VP16 first line treatment and response.**

|  | Carboplatin + Etoposide VP16 | Cisplatin + Etoposide VP16 |
| --- | --- | --- |
| First line chemotherapy |  |  |
| Patients, n | 543 | 279 |
| Number of cycles  Mean (sd)  Median [min-max] | 4.2 (1.8)  4 (1-9) | 4.7 (1.8)  5 (1-12) |
| Duration of treatment  Mean (sd), months  Median [min-max], months | 2.87 (1.78)  2.9 [0-15.1] | 3.11 (1.67)  3.5 [0-16.1] |
| Best response  Unknown  CR  PR  SD  PD  NE  NR | 51 (9.4%)  15 (2.8%)  274 (50.5%)  38 (7.0%)  79 (14.5%)  54 (9.9%)  32 (5.9%) | 32 (11.5%)  9 (3.2%)  155 (55.6%)  29 (10.4%)  24 (8.6%)  16 (5.7%)  14 (5.0%) |
| End of treatment, n (%) | 467 (86.0%) | 236 (84.6%) |
|  |  |  |
| Second line chemotherapy |  |  |
| Patients, n | 193 | 121 |
| Time from end of 1L to start of 2L  Mean (sd), months  Median [min-max], months | 4.42 (6.08)  3.10 [0-66.8] | 4.95 (5.54)  3.80 [0-37.3] |

CR, complete response; PR, partial response; sd, standard deviation; SD, stable disease; PD, progressive disease; NE, not estimated; ND, not determined.
