# Supplementary material for: Epidemiology, treatment, and survival in small cell lung cancer in Spain: Data from the Thoracic Tumor Registry
Source: PLoS One. 2021 Jun 2;16(6):e0251761. doi: 10.1371/journal.pone.0251761 (PMC8171958; doi:10.1371/journal.pone.0251761)
Supplement: S7 Table — CNS, central nervous system; ECOG, Eastern Cooperative Oncology Group. (DOCX) [file pone.0251761.s007.docx]

**S7 Table.** **Patient characteristics of long-term survivors (> 2 years; n = 63).**

| Characteristic | n | % |
| --- | --- | --- |
| **Sex**  Male  Female | 44  19 | 69.8  30.2 |
| **Age at diagnosis**  Mean (SD), years  Median [min-max], years | 63.5 (8.0)  63 [43-87] | |
| **Smoking habit**  Never smoker  Former smoker  Smoker | 3  28  32 | 4.8  44.4  50.8 |
| **ECOG at diagnosis**  0  1  ≥2 | 20  37  6 | 31.7  58.7  9.5 |
| **Metastasis at diagnosis**  Liver  Bone  Lung  Extrathoracic lymphadenopathy  Thoracic lymphadenopathy  Adrenal  Pleural effusion  CNS  Other | 60  19  15  14  13  12  12  8  6  6 | 95.2  30.2  23.8  22.2  20.6  19.0  19.0  12.7  9.5  9.5 |
| **First-line treatment**  No chemotherapy  Carboplatin + Etoposide VP16  Cisplatin + Etoposide VP16  Pemetrexed + Carboplatin  Vinorelbine + Cisplatin | 1  40  20  1  1 | 1.6  63.5  31.7  1.6  1.6 |

CNS, central nervous system; ECOG, Eastern Cooperative Oncology Group.
